# Bacterial Cellulose Containing Combinations of Antimicrobial Peptides with Various QQ Enzymes as a Prototype of an “Enhanced Antibacterial” Dressing: In Silico and In Vitro Data

**DOI:** 10.3390/pharmaceutics12121155

**Published:** 2020-11-27

**Authors:** Aysel Aslanli, Ilya Lyagin, Nikolay Stepanov, Denis Presnov, Elena Efremenko

**Affiliations:** 1 Faculty of Chemistry, Lomonosov Moscow State University, Lenin Hills 1/3, 119991 Moscow, Russia; ayselaslanli@mail.ru (A.A.); lyagin@mail.ru (I.L.); na.stepanov@gmail.com (N.S.); 2 N.M. Emanuel Institute of Biochemical Physics RAS, Kosygina str., 4, 119334 Moscow, Russia; 3 Faculty of Physics, Lomonosov Moscow State University, Lenin Hills 1/2, 119991 Moscow, Russia; denis.presnov@physics.msu.ru

**Keywords:** bacterial cellulose, combination, antimicrobial peptide, quorum-quenching enzymes, molecular docking, enhanced antibacterial activity, surface morphology, dressing prototype

## Abstract

To improve the action of already in use antibiotics or new antimicrobial agents against different bacteria, the development of effective combinations of antimicrobial peptides (AMPs) with enzymes that can quench the quorum (QQ) sensing of bacterial cells was undertaken. Enzymes hydrolyzing *N*-acyl homoserine lactones (AHLs) and peptides that are signal molecules of Gram-negative and Gram-positive bacterial cells, respectively, were estimated as “partners” for antibiotics and antimicrobial peptides in newly designed antimicrobial–enzymatic combinations. The molecular docking of six antimicrobial agents to the surface of 10 different QQ enzyme molecules was simulated in silico. This made it possible to choose the best variants among the target combinations. Further, bacterial cellulose (BC) was applied as a carrier for uploading such combinations to generally compose prototypes of effective dressing materials with morphology, providing good absorbance. The in vitro analysis of antibacterial activity of prepared BC samples confirmed the significantly enhanced efficiency of the action of AMPs (including polymyxin B and colistin, which are antibiotics of last resort) in combination with AHL-hydrolyzing enzymes (penicillin acylase and His_6_-tagged organophosphorus hydrolase) against both Gram-negative and Gram-positive cells.

## 1. Introduction

Bacterial cellulose (BC) is already considered an excellent exudate-removing wound dressing material that can be functionalized with some additives or modifications to avoid infections and reduce local pain [1,2,3]. Since BC possesses good absorptive characteristics, the simplest method for functionalizing it is the absorption of various chemicals into BC samples with further drying. Various antibiotics [4], polymers [5,6] or even whole antibiotic producers [7] can be used to impart antimicrobial properties to the dressings; however, the widespread use of antibiotics has led to a rise in resistant strains of pathogenic bacteria [8]. Thus, the development of new dressing materials should be based on modern approaches to the problem of bacterial resistance. One of these approaches is the use of antimicrobial agents in combination with enzymes providing quenching of the quorum sensing (QS) mechanism possessed by both Gram-positive (G(+)) and Gram-negative (G(−)) bacteria, which results in antibiotic resistance of their populations [9]. It is known that most G(−) bacteria commonly use multiple *N*-acyl homoserine lactones (AHLs) as QS inducers, whereas the main QS signaling molecules in G(+) bacteria are various autoinducing peptides (AIPs) [10,11]. Thus, the enzymatic hydrolysis of the signaling molecules of bacterial cells in combination with antimicrobial agents looks very attractive generally [12,13] and especially regarding dressing materials. Since we did not find related investigations that have been done previously, the design of BC samples containing combinations of different quorum-quenching (QQ) enzymes with antimicrobial agents became the main purpose of the work.

The most promising enzymes capable of hydrolyzing AHLs are considered to be: (i) lactonases, which break down the ester bond within the lactone ring, and (ii) acylases, which cleave the amide bond between the lactone ring and acyl substitute. For example, hexahistidine-tagged organophosphorus hydrolase (His_6_-OPH), which hydrolyzes various AHLs [14], belongs to the first group of enzymes. Its combination with various β-lactam antibiotics leads to an improvement of the action of both components [15,16,17]. Non-covalent enzyme–polyelectrolyte complexes of His_6_-OPH and poly(amino acids) developed for in vivo application [18,19] seem to be even more effective due to their stabilizing effect. A similar approach applied to His_6_-OPH and antimicrobial peptides (AMPs) led to the creation of combined antibacterial preparations, which increased both antimicrobial and catalytic activity [20,21]. However, the spectrum of AHLs is quite wide and diverse for a single enzyme (from C4 to C18, with or without 3-oxo substitution), and thus there is an interest in the development of dressing materials based on different AMPs and other enzymes which hydrolyze multiple AHLs. It is supposed that various anti-quorum preparations with high QQ enzymatic and antibacterial activity against pathogenic G(−) bacteria can form the basis for innovative antimicrobials that can be applied inside of dressings. At the same time, to the best of our knowledge, there is no information in the literature on the use of any enzymes or their combinations with antibacterial agents to inhibit QS in G(+) bacteria to date. However, some enzymes belonging to a large peptidase subclass could theoretically hydrolyze AIPs, and thus will be a perfect basis to combine with AMPs and to obtain effective antimicrobial agents against G(+) bacterial cells. The use of BC as a carrier for the absorption immobilization of different combinations of QQ enzymes with antimicrobial agents should also introduce novelty to the development of dressings.

Thus, among the main tasks of this work were not only the selection of the most effective combinations of antimicrobial agents with enzymes hydrolyzing QS signal molecules of G(−) or G(+) bacteria (AHLs or AIPs, respectively), but also to simulate computer models of the same “enzyme–antimicrobial” non-covalent complexes in silico and predict their possible physical–chemical characteristics. Such novel materials developed on the basis of BC in a dry form, and with a wide range antibacterial activity against both G(+) and G(−) bacterial cells, could be quite interesting for biopharmaceutics, veterinary medicine and other industries.

## 2. Materials and Methods

### 2.1. Materials

Polymyxin B, colistin and enzymes (penicillin acylase, thermolysin, carboxypeptidase A) were purchased from Sigma-Aldrich (Darmstadt, Germany). Indolicidin and temporin A were obtained from AnaSpec (Fremont, CA, USA). Recombinant *Escherichia coli* strain SG13009[pREP4] (Qiagen, Hilden, Germany) transformed by a plasmid encoding His_6_-OPH was used for the production of His_6_-OPH by a patented method and, further, the enzyme was purified by a published procedure [22].

Bacterial cellulose was produced with *Komagataeibacter xylinum* B-12429 cells, as was previously described for a medium with fructose [21,23], then dried at room temperature overnight under sterile conditions and cut to samples of 1 × 1 cm before further use.

The characteristics of the BC modified by the loading of AMP–enzyme combinations were the same as previously published [21]. The characteristics of all enzymes used in the work were controlled according to methods described earlier [24]: (i) protein concentration was determined by Bradford assay with Coomassie Brilliant Blue G-250; (ii) protein purity was analyzed by sodium dodecyl sulfate polyacrylamide gel electrophoresis in 12% polyacrylamide gel using a Mini-PROTEAN II cell (Bio-Rad, Hercules, CA, USA) followed by Coomassie Brilliant Blue R-250 staining; (iii) enzymatic activity was controlled by procedures traditionally used for the detection of QQ enzymes [21,25]. The purity of all used enzyme preparations applied in the work was ca. 99 ± 0.5%.

### 2.2. Antibacterial Combinations Based on Antibacterial Agents and Enzymes Loaded onto Bacterial Cellulose (BC)

All tested BC samples with combinations of antimicrobial agents and hydrolytic enzymes were prepared by the same general procedure.

Ten microliters of water solution of 0–1 g × L^−1^ antimicrobial agent and 5 μL of 1 g × L^−1^ enzyme solution in a 50 mM phosphate buffer (pH 7.4) containing 150 mM NaCl were applied to samples of bacterial cellulose (1 cm × 1 cm), and dried for 20–22 h at +8 °C under sterile conditions.

The antibacterial activity was analyzed according to the procedure described in [26], previously published for experiments with cells of the G(−) bacterium *Pseudomonas* sp. 78G (All-Russian Collection of Microorganisms, Russia), and the G(+) bacterium *Bacillus subtilis* B-522 (All-Russian Collection of Microorganisms, Russia). Cells were aerobically cultivated in Luria–Bertani (LB) culture medium on a thermostatically controlled Adolf Kuhner AG shaker (Basel, Switzerland) at 28 °C (for G(−)) and 30 °C (for G(+)), with stirring at 150 rpm. Cell growth was monitored with an Agilent UV-8453 spectrophotometer (Agilent Technology, Waldbronn, Germany) at 540 nm. Bacterial cells were grown for 18–20 h, and then separated from the culture broth by centrifugation at 8,000*g* for 10 min (Avanti J25, Beckman, Brea, CA, USA). Cell biomass was suspended in sterile 0.9% NaCl solution at a concentration of (1–2) × 10^8^ cells × mL^−1^.

Fifty microliters of a suspension of *Pseudomonas* sp. 78G or *B. subtilis* B-522 with a concentration of (1–2) × 10^8^ cells × mL^−1^ was loaded onto the BC samples. After 24 h of exposure, samples were placed in 1 mL of DMSO and gently stirred. Following 3 h of extraction, the residual concentration of ATP in the extract was determined using a standard luciferin–luciferase ATP reagent (Lyumtek Ltd., Moscow, Russia) by a known protocol [27,28]. BC samples containing only individual antimicrobial agents (i.e., polymyxin B, colistin, indolicidin and temporin A) without QQ enzymes were used as controls. The intensity of bioluminescence was measured using a Microluminometer 3560 (New Horizons Diagnostic, Arbutus, MD, USA). The calibration curves for the determination of cell concentrations were plotted, where the concentration of ATP was used as a function of the concentration of colony-forming units (CFU), calculated by a traditional microbiological method, using agar-containing media.

The effective concentrations of antimicrobials leading to a 50% decrease in the amount of living cells were assumed as values of *EC*_50_. The experiments were undertaken no less than in triplicate.

### 2.3. Water Absorption and Scanning Electron Microscopy (SEM) Analysis

To estimate the water absorption, BC samples were prepared as is mentioned above and placed in 0.9% NaCl with the addition of Coomassie Brilliant Blue G-250 to follow the change in color and weight of the samples over time. The hydration capacity was calculated by measuring the initial weight and the weight of the same sample after immersion in water solution for a defined period of time.

To make SEM images, BC samples were freeze-dried with a Freeze Dry System (Labconco, Kansas City, MO, USA), sectioned, sputtered with gold and studied with a Supra 40-30-87 microscope (Carl Zeiss, Oberkochen, Germany) at various magnifications.

### 2.4. Computational Methods

The I-TASSER server (ver. 4.4, available at http://zhanglab.ccmb.med.umich.edu/I-TASSER/) [29] was used to predict structures of AMPs and peptidases (Coccolysin, UniProt q833v7; Griselysin, UniProt a0a2x2lne8; Microcystinase, UniProt q93ca6; Mycolysin, UniProt p20910; Stearolysin (= thermolysin), UniProt p06874) as described earlier [20]. Briefly, the primary sequence of the polypeptide was uploaded to the server and its possible folded structures were generated using default options. Further, structures were aligned to the best one using the PyMOL Molecular Graphics System (ver. 1.7.6, Schrödinger Inc., New York, NY, USA). Usually, the first model was the most averaged and was used in modeling.

Crystallographic structures of lactonase AiiA from *Bacillus thuringiensis*, penicillin acylase PvdQ from *Pseudomonas aeruginosa* and carboxypeptidase A from bovine pancreas were obtained from the Protein Data Bank (PDB 4m1j, 2btn and 1yme, respectively). Based on the known structure of recombinant paraoxonase-1 (PON1, PDB 3srg), the structure of PON2 was predicted with the I-TASSER server, taking into account existing amino acid substitutions [30]. The His_6_-OPH dimer was obtained using the known crystallographic structure of OPH (PDB 1qw7), which was modified by His_6_-tag as described previously [20].

To calculate the surface charge distribution of enzymes and AMPs at a certain pH, the Adaptive Poisson–Boltzmann Solver (APBS) and PDB2PQR servers (ver. 1.4.2.1 and 2.1.1, respectively, available at http://www.poissonboltzmann.org/) with a PARSE force field and default settings were used [31,32]. After that, the structures in PQR format were converted to PDBQT format using AutoDockTools (as part of MGLTools ver. 1.5.6, available at http://mgltools.scripps.edu/) [33].

Enzyme–ligand complexes were calculated at the Supercomputing Center of Lomonosov Moscow State University [34], utilizing up to 512 cores of Intel Xeon X5570 2.93GHz and 1.5 TB of memory. Briefly, the Intel MPI Library (ver. 5.0.1), in addition to AutoDock Vina (ver. 1.1.2, available at http://vina.scripps.edu/) [35], was applied. The grid box was approximately centered on the center of mass of the enzyme. The size of the grid box was chosen so that any enzyme surface was within the box with an additional margin. Calculations were performed with default program options. Following the procedure, the “receptor” (i.e., enzyme) was proposed as rigid and the “ligand” (i.e., AMP) was fully flexible. The best six poses with minimal energy were selected.

The solvent-accessible area occupied by AMPs on the surface of the enzyme was calculated using the “*get_area*” function of PyMOL. Statistical analysis was realized using SigmaPlot (ver. 12.5, Systat Software Inc., San Jose, CA, USA), and the data are presented as means ± standard deviation (± SD) unless otherwise stated.

## 3. Results

### 3.1. Enzymes for Hydrolysis of G(−) Bacteria Signal Molecules

#### 3.1.1. Physical–Chemical Interactions in Simulated Models of Enzyme–AMP Combinations

The following enzymes with different AHL-hydrolyzing activity were selected based on a review of the literature for this work: lactonase from *Bacillus thuringiensis* (AiiA, PDB 2btn), penicillin acylase from *Pseudomonas aeruginosa* (PvdQ, PDB 4m1j) and recombinant human paraoxonase-2 (PON2). The main criteria for selection were the following: activity at neutral or weak alkaline pH values, substrate specificity and stability at mild temperatures and at temperate ionic strength. The substrate specificity of all selected enzymes (in addition to His_6_-OPH) varied significantly for structurally different AHLs [36] and could be multiplied in a single combined preparation. The structure of PON2 has not been experimentally solved yet, and thus, it was predicted by us based on the current structure of PON1, taking into account the known amino acid replacements in the enzyme [30].

The following antimicrobial agents were used in the work: polymyxin B, polymyxin E (= colistin) (both were taken as effective antibiotics of a polypeptide nature considered as “antibiotics of last resort”), dermicidin, oritavancin, indolicidin and temporin A (all were taken as prospective peptides, possessing wide enough antimicrobial activity) [37].

**Figure 1:**
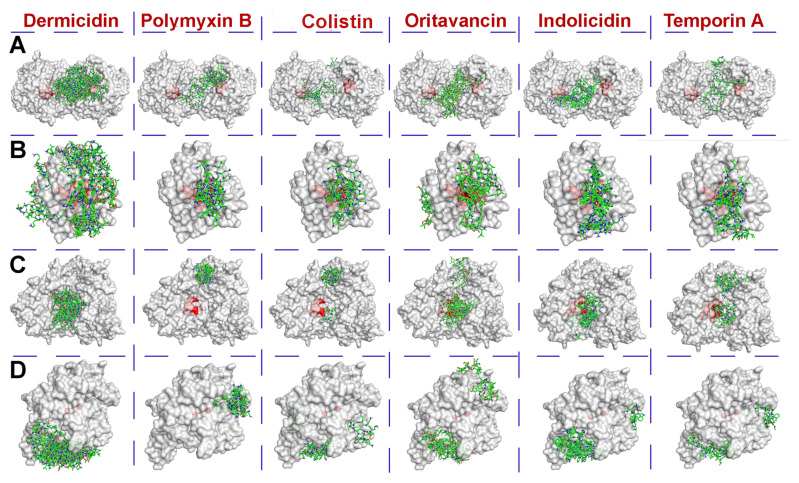
The calculated localization of dermicidin, polymyxin B, colistin, oritavancin, indolicidin and temporin A on the molecules of a hexahistidine-tagged organophosphorus hydrolase (His_6_-OPH) dimer (**A**), lactonase 2btn (**B**), acylase 4m1j (**C**) and predicted PON2 (**D**). The active site area of enzymes is colored red, while molecules of antimicrobial agents are colored green.

Using the molecular docking method of computer modeling, possible interaction modes of a number of antimicrobial agents with a polypeptide nature were calculated with molecules of selected enzymes at pH 7.5, which is closest to the physiological one (Figure 1). Analysis of the binding energy values (Table 1) showed that oritavancin possessed the strongest affinity to all enzymes.

The maximal strength of non-covalent interaction was revealed for the oritavancin–acylase complex. Indolicidin could also be noticed among other AMPs with relatively high levels of binding to molecules of different enzymes.

It should be noted that structures of colistin and polymyxin B differ from each other only by one amino acid within the cyclic part at position 6 (Phe in polymyxin B and Leu in colistin). Nevertheless, on average, polymyxin B was found to bind to the enzymes a little more strongly than colistin. According to a one-way ANOVA of binding energy values (N = 6), for each antimicrobial agent, there was a statistically significant difference in all groups (*p* < 0.05); pairwise multiple comparisons are presented in Table S1.

The area occupied by tested antimicrobial agents (Table 1) weakly correlated with their affinity to the molecule surface of different enzymes. On average, polymyxin B and colistin occupied the smallest area both on the total surface of enzymes and near their active sites.

Overall, the pair PON2 and His_6_-OPH possessed similar good characteristics among enzymes, while lactonase was a notable outsider. Penicillin acylase seems to be quite sensitive and accepts only polymyxins from all the studied antimicrobial agents. Nevertheless, it was selected together with His_6_-OPH for further in vitro studies.

#### 3.1.2. Antimicrobial Activity of AMP Combinations with AHL-Hydrolyzing Enzymes

**Figure 2:**
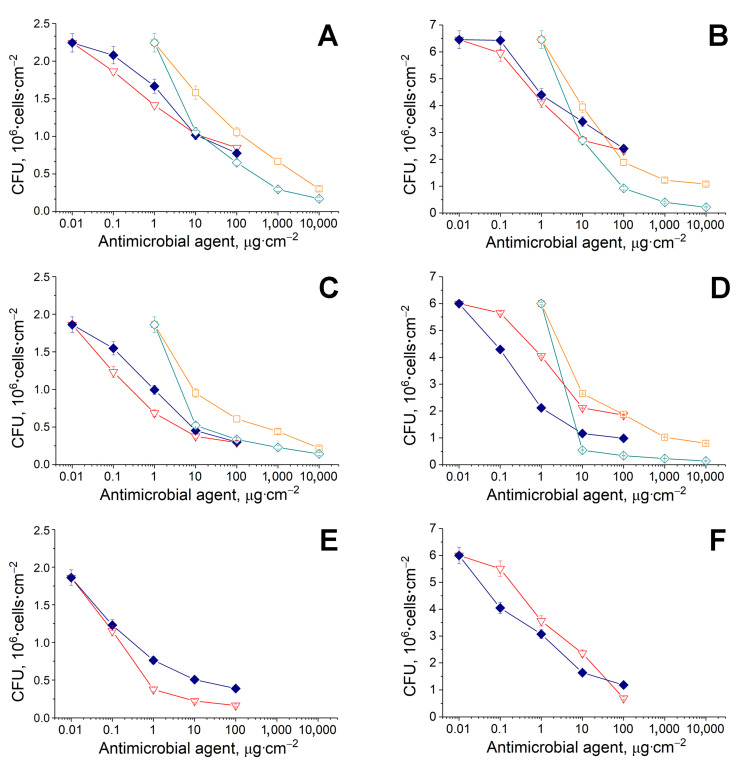
Antimicrobial activity of colistin (◆, blue), polymyxin B (▽, red), indolicidin (□, orange) and temporin A (◇, cyan) alone (**A**,**B**) or in complex with His_6_-OPH (**C**,**D**) or penicillin acylase (**E**,**F**). The *B. subtilis* B-522 (**A**,**C**,**D**) or *Pseudomonas* sp. 78G (**B**,**D**,**F**) cells were loaded on each sample of BC (1 cm^2^), containing a certain antimicrobial agent, and exposed to obtain a dose-dependent manner of cell death.

Since polymyxin B, colistin, indolicidin and temporin A left after molecular docking for further investigation, the sterile samples of BC, obtained with known characteristics by using immobilized bacterial cells and a previously developed procedure for BC production [23], were loaded with several combinations of the AMPs with His_6_-OPH or penicillin acylase for in vitro experiments. The antibacterial activity of BC samples with antimicrobial agents in the presence and absence of enzymes was determined, applying G(+) (*Bacillus subtilis*) and G(−) (*Pseudomonas* sp.) cells (Figure 2).

Before cell suspensions’ loading, all variants of tested antibacterials (AMPs with or without AHL-hydrolyzing enzymes) were deposited onto the BC samples (1 cm^2^) and dried. Then, all BC samples with bacteria were exposed under hermetic conditions, which led to cell death in a dose-dependent manner.

The G(+) cells were used in these experiments because the AMPs possess wide enough antibacterial activity, and it was interesting to evaluate the maintenance of their wide action ability against both G(−) and G(+) bacteria in combinations with AHL-hydrolyzing enzymes.

Polymyxin B and colistin, loaded onto BC samples with His_6_-OPH or penicillin acylase, showed noticeably better antimicrobial activity towards both G(+) and G(−) cells as compared to the AMPs without the AHL-hydrolyzing enzymes (Figure 2, Table 2).

In the case of G(+) cells, the general tendencies in action of the two AMPs continued to be the same in the presence of both His_6_-OPH and penicillin acylase as it was without the enzymes, whereas in the case of G(−) cells, colistin started to become considerably more pronounced than polymyxin B in the presence of the QQ enzymes as compared to the situation without them. The effective concentrations of colistin (*EC*_50_) (Table 2), which are required for a 50% reduction in the number of G(−) cells, were significantly lower when the AMP was applied in BC samples in combination with the AHL-hydrolyzing enzymes.

Indolicidin and temporin A, when added to BC samples, affected both G(+) and G(−) cells. Generally, the values of *EC*_50_, determined for indolicidin and temporin A, were comparable with those estimated for polymyxin B and colistin, known as reserve antibiotics [38]. According to a one-way ANOVA of *EC*_50_ values, there was a statistically significant difference in all groups (*p* < 0.01); pairwise multiple comparisons are presented in Table S2.

This result seems very attractive, because it allows us to consider the “new” AMPs as good enough candidates for their use as effective antimicrobial agents, but their application looks more advisable against G(−) bacterial cells.

Overall, temporin A was slightly more effective than indolicidin in action against both types of cells, and this tendency did not change in the presence of His_6_-OPH. However, the application of indolicidin and temporin A in combination with the enzyme certainly resulted in, respectively, a 4.5–5.5-fold and 1.5–1.9-fold lowering of their effective concentrations (*EC*_50_) necessary for the cell death of both G(+) and G(−) cell types. At the same time, the combinations of indolicidin and temporin A with penicillin acylase were not “productive” (there was no positive or negative influence as compared to control with AMPs alone) (data not shown).

These results confirmed the conclusions made after computer modeling. It appeared that His_6_-OPH can be successfully combined with a larger number of AMPs as compared to penicillin acylase, improving their antimicrobial action, for use in the development of dressing prototypes. However, it was shown that the action of known AMPs, such as colistin and polymyxin B, will be more attractive and efficient as compared to novel AMPs (indolicidin and temporin A), since their effective concentrations, which are required for the death of various cells, were lower, especially in combination with AHL-hydrolyzing enzymes.

### 3.2. Enzymes for Hydrolysis of G(+) Bacteria Signal Molecules in Combination with AMPs

#### 3.2.1. Physical–Chemical Interactions in Simulated Models of AM Combinations with Peptidases

**Figure 3:**
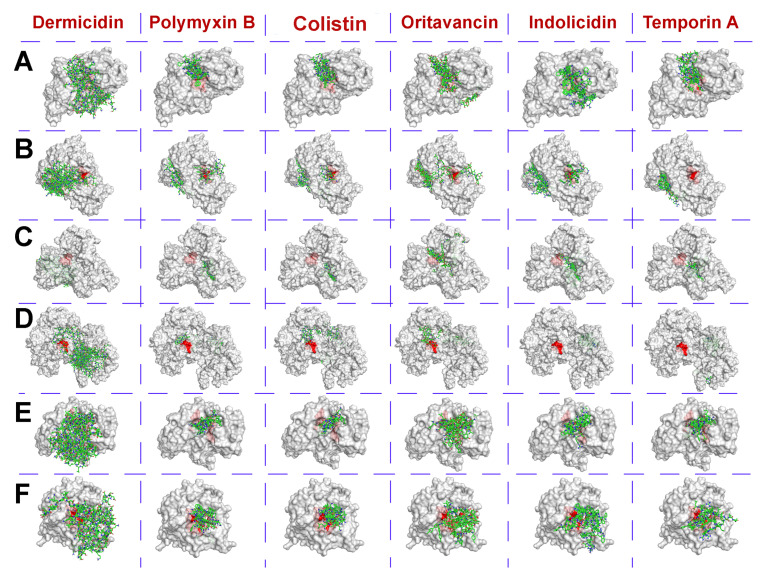
The calculated localization of dermicidin, polymyxin B, colistin, oritavancin, indolicidin and temporin A on the surface of coccolysin (**A**), griselysin (**B**), stearolysin (**C**), mycolysin (**D**), microcystinase (**E**) and carboxypeptidase A (**F**). The active site area of enzymes is colored red, while AMP molecules are colored green.

Several peptidases that are active under physiological conditions and are potentially capable of hydrolyzing different AIPs of G(+) bacterial cells were selected with criteria similar to those previously used for AHL-hydrolyzing enzymes (Table 3). Their interaction models with AMPs, possessing wide enough antibacterial activity and tested previously with AHL-hydrolyzing enzymes, were simulated with the same procedure (Figure 3).

It was shown that antimicrobial agents can bind to the peptidases in a similar way, which was previously determined for AHL-hydrolyzing enzymes (Table 4). The strongest bond was observed for oritavancin and indolicidin. The highest affinity value for most AMPs was found in complexes with stearolysin (= thermolysin).

Polymyxin B bound to most peptidases more strongly than colistin and temporin A. The least durable was the interaction of peptidases with dermicidin. According to a one-way ANOVA of binding energy values (N = 6) for each antimicrobial agent, there was a statistically significant difference in all groups (*p* < 0.05); pairwise multiple comparisons are presented in Table S3.

On average, AMPs occupied the largest area (ca. 18–19%) on the surface of griselysin (Table 4). The maximum occupied area (up to 36%) was observed for complexes of dermicidin with coccolysin and carboxypeptidase A.

The greatest burying of active sites was found in the case of carboxypeptidase A, which could indicate the presence of some enzymatic activity with this peptide studied in silico.

The best situation was in the case of thermolysin, where AMPs totally occupied relatively small areas and had almost “zero” localization near its active site.

Thus, dermicidin turned out to be an outsider among the studied antimicrobial agents for effective complexing with peptidases.

Polymyxin B, colistin and temporin A are likely to be a middle ground, especially when coupled with thermolysin. It was concluded that carboxypeptidase A can be selected as the opposite example to thermolysin (i.e., “negative” control) for further experiments in vitro with bacterial cells.

#### 3.2.2. Antimicrobial Activity of BC Samples Loaded with AMPs in Combination with Peptidases

**Figure 4:**
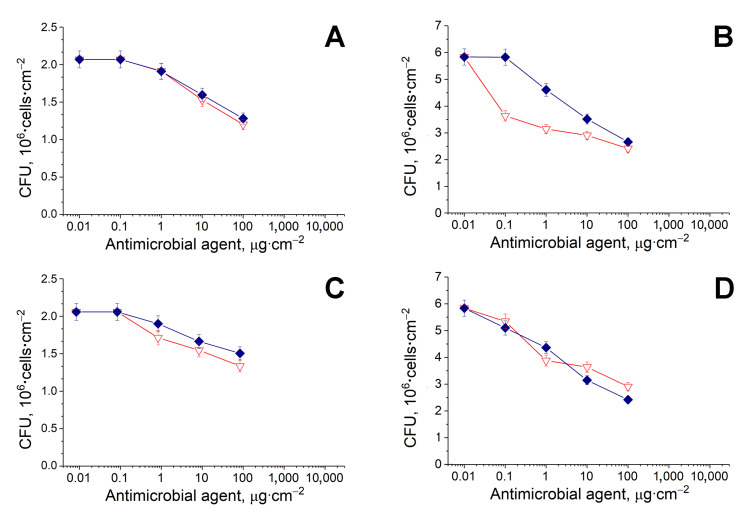
Antimicrobial activity of colistin (◆, blue) and polymyxin B (▽, red) in combination with thermolysin (**A**,**B**) or carboxypeptidase A (**C**,**D**). The *B. subtilis* B-522 (**A**,**C**) or *Pseudomonas* sp. 78G (**B**,**D**) cells were loaded on a sample of BC (1 cm^2^) containing an antimicrobial, and exposed for 24 h, which led to their death in a dose-dependent manner.

The antimicrobial activity of antibacterials with or without thermolysin or carboxypeptidase A was determined after their loading onto BC samples (Figure 4, Table 2). It was revealed (Figure 4) that the action efficiency of polymyxin B and colistin (i.e., the most effective ones), in combination with peptidases, was markedly worsened, especially against *B. subtilis* cells (Table 2) as compared to the effect of the AMPs in controls without any QQ enzymes (Figure 2A,B). Surprisingly, polymyxin B and colistin, in combination with peptidases, demonstrated even more effect on G(−) cells in comparison with the results demonstrated by the same combinations in relation to G(+) cells, where peptidases should perform their catalytic activity against AIPs.

Thus, the non-covalent interaction of AMPs with peptidases, predicted by molecular docking, was absolutely negative for the antibacterial activity of both tested AMPs against G(+) cells and gave unpredicted results for the combination of thermolysin with polymyxin B in experiments with *Pseudomonas* sp. cells, when the value of *EC*_50_ appeared to be lower compared to polymyxin B alone. In the case of G(−) cells, the thermolysin probably played the role of a “carrier” for the AMP and modified its confirmation, owing to inter-peptide binding, and thereby improved its antibacterial activity.

### 3.3. Absorption Capacity of BC Samples

**Figure 5:**
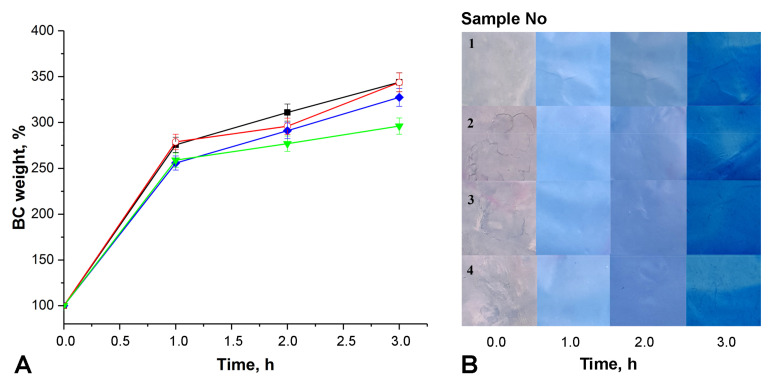
Kinetics absorption of 0.9% NaCl solution with the addition of Coomassie Brilliant Blue G-250 by dry BC samples ((**A**) weight changing, (**B**) visual observation of color intensity changing) without any loadings (⯀—sample #1), and loaded with colistin (**○**—sample #2), colistin in combination with His_6_-OPH (◆—sample #3) or penicillin acylase (▼—sample #4).

The possible changes in the absorption capacity of BC samples containing combinations of AMPs with His_6_-OPH or penicillin acylase as compared to BC samples without “antimicrobial” loadings were specially investigated. All fiber samples were tested, but only some of them, which seemed to us as the most interesting from the applied point of view, are presented as examples of undertaken experiments in Figure 5.

The BC samples with the most “fruitful” combinations of penicillin acylase and His_6_-OPH with colistin and polymyxin B were used to assess their absorption properties simultaneously with unmodified BC. The absorption kinetics of colored 0.9% NaCl solution with dry BC samples showed great similarity in the overall process trend between controls and all the tested variants. The initial velocity of water sorption appeared to be the same regardless of the combinations of AMPs with QQ enzymes loaded on BC samples. A significant increase in the weight of BC samples was observed during the first hour, and then it continued to grow for several hours, but with half as much absorption intensity. Actually, the observed tendency towards absorption was in general typical for pure dry BC samples, as is known from the literature [4].

At the same time, some differences were revealed between the samples modified by different combinations of AMPs with QQ enzymes: the kinetics of water absorbance was “a little delayed” in the BC with enzymes. However, observation of the samples did not make it possible to visually distinguish this difference during the first 3 h (Figure 5B). Nevertheless, this difference can be taken into account in further in vivo experiments, especially in the case of the prolonged use of BC dressings with similar combinations of antibacterials.

### 3.4. Scanning Electron Microscopy of the Surface of BC Samples

**Figure 6:**
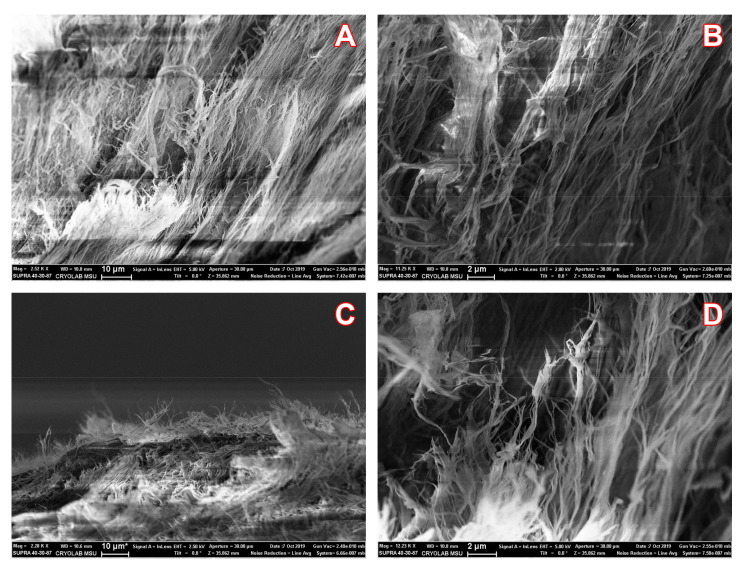
The SEM images of fiber surface morphology of dry BC samples before (**A**,**B**) and after (**C**,**D**) loading of colistin in combination with His_6_-OPH.

Since some combinations of AMPs with QQ enzymes were loaded onto BC samples, it was interesting to use scanning electron microscopy (SEM) to visualize the surface of the dry material, which is usually important for dressings, and to assess the possible appearance of some notable changes in the materials. SEM of BC with a combination of colistin with His_6_-OPH is given as an example of the results obtained, which were generally very similar to each other (Figure 6).

The images obtained clearly confirmed the presence of a nanofibrillar structure in the analyzed BC samples without noticeable differences between those which were treated by developed combinations or those without them. The “antibacterial” BC samples, visually retaining their texture without fiber clumping, theoretically provided absorption similar to native BC samples. So, the visual characteristics of the analyzed samples corresponded to the data in Figure 5.

## 4. Discussion

Several criteria, as are known from previous studies [17,18,20,21], are important for the successful computational selection of partners for combination with enzymes. The most crucial ones are steric hindrances for substrate entrance into the enzymatic active center, as binding energy (affinity) of these partners to enzyme surface. For example, the minimization of both parameters could lead to active and stable enzyme non-covalent complexes with partners that are polymers [20]. A balance is needed for the complexes of enzymes with antibiotics that are polypeptides. If such complexes are too durable, the bioavailability of the antimicrobial agent will be decreased. Additionally, vice versa, the bioavailability will be unchanged (or minimally disturbed) for weak complexes, while components are highly likely to be separated from each other. Thus, this is also undesirable.

The search for potentially effective combinations of AMPs with QQ enzymes using computer simulations predicted positive results and prevented overtly negative wet experiments. For example, oritavancin and dermicidin have shown exactly these extreme affinities in the current work (Table 1 and Table 4). Therefore, both of these AMPs were identified as unsuitable for combination with the chosen QQ enzymes, though they could be interesting for other studies. Indolicidin appeared to be very close to oritavancin and was shown here with temporin A only for comparison with previous results [21].

Interestingly, even a single amino acid modification in the structure of an AMP can affect its complexing with an enzyme. It turned out that, in general, polymyxin B binds to QQ enzymes a little more strongly than colistin (= polymyxin E). Both polymyxin B and colistin occupied minimal enzymatic surfaces and were located far from active centers, and generally seemed the most promising AMPs to combine with various QQ enzymes from the experiments in silico.

The usual and widely distributed bacteria of the *Pseudomonas* and *Bacillus* genera were selected for in vitro experiments. Their closest relatives are *Pseudomonas aeruginosa*, known to be predominantly dependent on alkyl-quinolone (through PqsR) and AHL (through TraR) QS, and *Bacillus cereus*, which depends on AIPs (through PlcR). Thus, both AHLs and AIPs were equally represented here.

The in vitro testing of the antimicrobial action of the combined preparations revealed some unexpected results. When combining polymyxin B and colistin with AHL-hydrolyzing enzymes, their antibacterial activity increased by several times (Figure 2, Table 2). The best compositions introduced into BC were the pairs penicillin acylase and colistin and His_6_-OPH and colistin, which ensured 12.5–13-fold (for both G(+) and G(−) cells) and 35-fold (for G(−) cells) decreases in *EC*_50_ values, respectively (Table 2). However, their antimicrobial activity deteriorated dramatically in combination with peptidases (Figure 4, Table 2).

Based on the molecular docking prediction and known properties of the AHL-hydrolyzing enzymes, their expected effectivity of action on G(−) cells was shown. However, it appeared that same enzymes improve the known action of the AMPs against G(+) cells in non-covalent combinations with the antimicrobial agents. Thus, significantly “enhanced” variants of the antibiotics of “last resort” were obtained in the work.

Both thermolysin and carboxypeptidase A might theoretically catalyze amide bond cleavage within AMPs, though, in toto, such cyclic peptides are considered to be more proteolytically stable as compared to those with a linear structure [39]. However, as it was positively shown for the combination of thermolysin with polymyxin B (Table 2), such peptidases can still be useful for the “enzymatic advance” and action improvement of antimicrobial agents, which may be the subject of further studies.

Interestingly, these enzymes have a quite similar specificity: thermolysin is specific for hydrophobic residues in the inner sequence part, while carboxypeptidase A is specific for C-terminal hydrophobic residues. Both polymyxin B and colistin have a single N-terminal residue of a noncanonical amino acid (α,γ-diaminobutanoic acid) modified by an acyl substituent (5-methylheptanoic acid) at the α-position. The possibility of such catalytic activity may be of some interest not only in terms of the resistance of microorganisms, but also in terms of metabolic transformation by animals, and may be the subject of separate studies.

Interestingly, those relatively new AMPs, such as indolicidin and temporin A, which have no current pharmaceutic application, demonstrated good enough values of *EC*_50_ in combination with His_6_-OPH; the values were comparable in activity against both G(−) and G(+) cells with those that are “the last resort”. Moreover, these results were reached in experiments with antibacterials immobilized on BC samples, when the mobility of the antimicrobial agents in relation to cells was reduced by the presence of the matrix. In solutions, the results might be even more pronounced.

It should be emphasized that in the preparation of the discussed effective combinations of QQ enzymes with already commercially produced antibiotics, it will only be necessary to mix them, and that is much easier as compared to the new chemical synthesis of polymyxin derivatives proposed as alternatives to enhance antibiotics [40].

Due to its good absorption capacity, BC is regarded as a suitable material for absorbing wound exudate and supporting healing as a prospective dressing material [1]. There are several known investigations of BC with different absorbed antibiotics [1,2,3,4,5], but none of them was focused on the spectrum studied herein, especially in combination with various QQ enzymes.

Of course, the introduction of enzymes into the content of human dressings should consider the immunogenic characteristics of the enzymes as foreign proteins. According to our previous investigations with His_6_-OPH, introduced into non-covalent complexes with different non-antimicrobial polypeptides [18], we can suppose that the appearance multiple intermolecular bonds should lead not only to the stabilization of the enzyme, but also to a decrease in its immunogenicity. However, this should be further confirmed with in vivo testing.

## 5. Conclusions

Thus, a computational rational design of multitarget antibacterial agents with improved characteristics was an extremely useful procedure in this investigation. Due to it, polymyxin B and colistin were predicted to have the greatest potential for combination with AHL-hydrolyzing enzymes, which was confirmed experimentally. The loading of AMP–QQ enzyme combinations onto BC samples allowed us to demonstrate in vitro the potential in the development of prototypes for antibacterial dressings with improved action of antimicrobials.

Taking into account the high absorption capacity of the obtained “antibacterial” BC samples, it is possible to think that they can be certainly attractive as prototypes of dressing materials for emergency use, with their subsequent short-term (after several hours) replacement due to a possible notable increase in the weight of the dressing itself. Since the antibiotics work significantly better in the revealed combinations with the chosen enzymes in relation both G(−) and G(+) cells, therefore, with a short-term application of the bandage, it will probably be sufficient not only to effectively absorb exudate from the wound, but also to prevent the possible development of contamination.

## Figures and Tables

**Table 1:** The calculated values of the binding energy (affinity) of antimicrobial agents with different *N*-acyl homoserine lactone (AHL) hydrolases, and the area occupied by the antimicrobial peptides (AMPs) on the surface of molecules of these enzymes. The values are coded by color palette, from red (minimal values in the group) to blue (maximal values in the group).

Antimicrobial Agent	Enzyme	Affinity, kJ × mol^−1^		Occupied Area, %	
		Mean	Median	Total	Near Active Sites
Dermicidin	Acylase	−20.4	−20.1 ± 0.9	13.5	0.6
	Lactonase	−14.6	−15.1 ± 1.0	30.7	0.7
	PON2	−14.7	−14.6 ± 0.5	16.8	0
	His_6_-OPH	−15.2	−15.1 ± 0.9	10.0	0.1
Polymyxin B	Acylase	−25.3	−24.7 ± 1.5	3.0	0
	Lactonase	−28.6	−27.8 ± 1.9	11.8	0.5
	PON2	−25.9	−26.1 ± 1.0	7.6	0
	His_6_-OPH	−23.0	−22.6 ± 1.1	7.6	0.4
Colistin	Acylase	−25.2	−24.5 ± 1.4	7.2	0.2
	Lactonase	−22.9	−22.8 ± 1.4	12.9	0.5
	PON2	−19.5	−19.2 ± 0.8	16.3	0
	His_6_-OPH	−21.5	−21.3 ± 1.0	9.6	0.1
Oritavancin	Acylase	−41.6	−41.8 ± 1.5	11.4	0.6
	Lactonase	−35.5	−34.9 ± 2.2	18.9	0.6
	PON2	−29.4	−29.1 ± 0.7	20.1	0
	His_6_-OPH	−33.7	−33.5 ± 0.9	7.5	0.1
Indolicidin	Acylase	−32.4	−32.2 ± 1.0	10.2	0.5
	Lactonase	−31.7	−31.6 ± 1.2	15.1	0.7
	PON2	−28.7	−28.2 ± 1.0	19.5	0
	His_6_-OPH	−32.3	−32.0 ± 1.2	7.8	0.6
Temporin A	Acylase	−25.3	−25.3 ± 0.4	9.2	0.6
	Lactonase	−26.1	−25.9 ± 0.6	15.8	0.7
	PON2	−26.6	−26.6 ± 0.5	16.5	0
	His_6_-OPH	−25.2	−25.1 ± 0.2	9.6	0.1

**Table 2:** The effective concentrations of antimicrobials resulting in a 50% decrease in cell amount (*EC*_50_, pg^.^cell^−1^) in the absence or presence of quorum-quenching (QQ) enzymes loaded onto bacterial cellulose (BC) samples.

**G(+) Bacterial Cells:** ***Bacillus subtilis*** **B-522**				
**QQ Enzyme**	**Colistin**	**Polymyxin B**	**Indolicidin**	**Temporin A**
None	6.16 ± 0.19	5.13 ± 0.16	66.72 ± 0.43	7.90 ± 0.06
His_6_-OPH	1.41 ± 0.03	0.38 ± 0.01	12.24 ± 0.23	5.31 ± 0.01
Penicillin acylase	0.47 ± 0.03	0.21 ± 0.01		
Thermolysin	614 ± 13	364 ± 12		
Carboxypeptidase A	14,166 ± 94	1684 ± 53		
**G(−) Bacterial Cells: *Pseudomonas* sp. 78G**				
None	4.61 ± 0.22	4.35 ± 0.11	6.93 ± 0.18	2.24 ± 0.03
His_6_-OPH	0.13 ± 0.02	1.16 ± 0.16	2.61 ± 0.24	1.18 ± 0.02
Penicillin acylase	0.37 ± 0.02	0.97 ± 0.02		
Thermolysin	17.28 ± 0.49	3.14 ± 0.32		
Carboxypeptidase A	7.05 ± 0.19	33.28 ± 1.39		

**Table 3:** The main characteristics of peptidases selected for investigation.

Peptidase *(Source)* [39]	Molar Mass; Optimal pH	* Preferable Peptide Bond for Cleavage
Coccolysin *(Enterococcus faecalis)*	31.5 kDa; pH 6–8	P1’ = Leu, Phe, Ile or Ala
Griselysin *(Streptomyces griseus)*	35.1 kDa; pH ~7	P1, P1’ = hydrophobic residues
Stearolysin *(Geobacillus stearothermophilus)*	34 kDa; neutral pH	P1, P1’ = hydrophobic residues
Mycolysin *(Streptomyces* sp*.)*	37.1 kDa; pH 7–8.5	P1’ = hydrophobic residues
Microcystinase *(Sphingomonadaceae* sp*.)*	36 kDa; pH 6.5–8.4	Arg–Adda bond **
Carboxypeptidase A *(bovine pancreas)*	34.6 kDa; pH 7–9	C-terminal hydrophobic residues (incl. acylated ones)

* The scissile peptide bond is located between residues P1–P1’, where P1 corresponds to the N-terminal group, and P1’ is the C-terminal group. ** Adda = 2*S*,3*S*,8*S*,9*S*-3-amino-9-methoxy-2,6,8-trimethyl-10-phenyldeca-4*E*,6*E*-dienoic acid.

**Table 4:** The calculated values of the binding energy (affinity) of AMPs with different peptidases and the area occupied by such AMPs on the surface of these enzymes. The values are coded by color palette, from red (minimal values in the group) to blue (maximal values in the group).

Antimicrobial Agent	Enzyme	Affinity, kJ × mol^−1^		Occupied Area, %	
		Mean	Median	Total	Near Active Sites
Dermicidin	Coccolysin	−14.4	−14.2 ± 0.6	32.5	0.1
	Griselysin	−13.1	−13.0 ± 0.6	16.4	0
	Stearolysin	−16.4	−16.3 ± 0.3	10.8	0
	Mycolysin	−12.3	−12.3 ± 0.5	14.8	0.7
	Microcystinase	−13.4	−13.4 ± 1.0	21.7	0
	Carboxypeptidase A	−18.7	−18.4 ± 1.2	35.6	1.9
Polymyxin B	Coccolysin	−29.1	−29.1 ± 1.5	9.1	0.3
	Griselysin	−30.5	−30.3 ± 1.0	17.3	0.2
	Stearolysin	−35.0	−35.1 ± 0.6	6.9	0
	Mycolysin	−32.3	−32.2 ± 1.4	10.8	0.4
	Microcystinase	−26.8	−26.8 ± 0.5	11.0	0.5
	Carboxypeptidase A	−26.3	−26.6 ± 2.1	13.5	1.9
Colistin	Coccolysin	−27.5	−27.2 ± 1.4	9.5	0.3
	Griselysin	−28.2	−27.6 ± 1.9	18.8	0.2
	Stearolysin	−31.7	−31.6 ± 1.2	5.4	0
	Mycolysin	−26.3	−26.1 ± 0.6	12.6	0.5
	Microcystinase	−27.6	−27.0 ± 2.1	10.3	0.6
	Carboxypeptidase A	−21.7	−21.7 ± 0.9	7.6	1.9
Oritavancin	Coccolysin	−39.0	−38.5 ± 1.7	15.2	0.4
	Griselysin	−39.0	−38.9 ± 1.9	18.9	0.2
	Stearolysin	−47.4	−47.1 ± 2.5	11.2	0.1
	Mycolysin	−34.7	−34.3 ± 1.0	10.8	0.5
	Microcystinase	−38.0	−37.6 ± 0.5	13.9	0.2
	Carboxypeptidase A	−36.7	−36.6 ± 1.6	11.2	1.7
Indolicidin	Coccolysin	−36.3	−36.6 ± 1.1	12.0	0.4
	Griselysin	−29.3	−29.3 ± 0.9	18.6	0.3
	Stearolysin	−43.9	−43.7 ± 0.8	10.2	0
	Mycolysin	−33.4	−33.0 ± 0.9	6.9	0
	Microcystinase	−36.8	−36.8 ± 0.8	13.0	0.6
	Carboxypeptidase A	−30.5	−30.1 ± 1.3	14.0	1.9
Temporin A	Coccolysin	−26.3	−26.3 ± 1.4	9.8	0.3
	Griselysin	−26.6	−26.8 ± 0.4	11.4	0
	Stearolysin	−29.8	−29.9 ± 0.6	9.2	0
	Mycolysin	−29.5	−29.5 ± 1.6	8.7	0
	Microcystinase	−31.5	−31.4 ± 1.1	11.6	0.6
	Carboxypeptidase A	−27.6	−27.6 ± 0.8	12.1	1.9

## Supplementary Materials

Supplementary Materials can be found at https://www.mdpi.com/1999-4923/12/12/1155/s1, Table S1: p-values of pairwise multiple comparisons (Holm–Sidak method) after one-way ANOVA of the binding energies (N = 6) of AMPs with different AHL hydrolases, Table S2: p-values of pairwise multiple comparisons (Holm–Sidak method) after one-way ANOVA of *EC*_50_ values with different bacteria, Table S3: p-values of pairwise multiple comparisons (Holm–Sidak method) after one-way ANOVA of the binding energies (N = 6) of AMPs with different peptidases.

## Abbreviations

AHL	*N*-acyl homoserine lactone
AIP	Autoinducing peptide
AMP	Antimicrobial peptide
BC	Bacterial cellulose
CFU	Colony-forming unit
His_6_-OPH	Hexahistidine-tagged organophosphorus hydrolase
QQ	Quorum quenching
PON2	Paraoxonase 2
