# Supplementary material for: Bacterial Cellulose Containing Combinations of Antimicrobial Peptides with Various QQ Enzymes as a Prototype of an “Enhanced Antibacterial” Dressing: In Silico and In Vitro Data
Source: Pharmaceutics. 2020 Nov 27;12(12):1155. doi: 10.3390/pharmaceutics12121155 (PMC7760664; doi:10.3390/pharmaceutics12121155)
Supplement: Supplementary file 1 [file pharmaceutics-12-01155-s001.pdf]

pharmaceutics

MDPI

Supplementary Materials: Bacterial Cellulose Containing Combinations of Antimicrobial Peptides with Various QQ Enzymes as a Prototype of an "Enhanced Antibacterial" Dressing: In Silico and In Vitro Data

Aysel Aslanli, Ilya Lyagin, Nikolay Stepanov, Denis Presnov and Elena Efremenko *

Table S1. p-values of pairwise multiple comparisons (Holm-Sidak method) after one-way ANOVA of the binding energies (N=6) of AMPs with different AHL hydrolases (Table 1). n.s. – statistically not significant (i.e. p>0.05).

Antimicrobial agent	Enzyme	*vs.* Acylase	*vs.* Lactonase	*vs.* PON2
Dermicidin	Lactonase	<0.001 (t = 12.081)		
	PON2	<0.001 (t = 11.793)	n.s.	
	His_6-OPH	<0.001 (t = 12.538)	n.s.	n.s.
Polymyxin B	Lactonase	0.001 (t = 4.197)		
	PON2	n.s.	0.005 (t = 3.483)	
	His_6-OPH	0.004 (t = 3.403)	<0.001 (t = 8.250)	0.001 (t = 4.228)
Polymyxin E	Lactonase	0.004 (t = 3.413)		
	PON2	<0.001 (t = 8.482)	<0.001 (t = 5.069)	
	His_6-OPH	<0.001 (t = 6.330)	0.024 (t = 2.389)	0.006 (t = 3.464)
Oritavancin	Lactonase	<0.001 (t = 7.285)		
	PON2	<0.001 (t = 14.488)	<0.001 (t = 7.203)	
	His_6-OPH	<0.001 (t = 9.438)	0.044 (t = 2.153)	<0.001 (t = 5.050)
Indolicidin	Lactonase	n.s.		
	PON2	<0.001 (t = 5.837)	<0.001 (t = 4.756)	
	His_6-OPH	n.s.	n.s.	<0.001 (t = 5.620)
Temporin A	Lactonase	0.032 (t = 2.812)		
	PON2	<0.001 (t = 4.857)	n.s.	
	His_6-OPH	n.s.	0.024 (t = 3.068)	<0.001 (t = 5.113)

Table S2. p-values of pairwise multiple comparisons (Holm-Sidak method) after one-way ANOVA of EC_{50} values with different bacteria (Table 2). n.s. – statistically not significant (i.e. p>0.05).

Bacterial target	Antimicrobial Agent	Enzyme	*vs.* None	*vs.* His$_6$-OPH	*vs.* Penicillin Acylase	*vs.* Thermolysin
Bacillus subtilis	Polymyxin E	His$_6$-OPH	n.s.			
		Penicillin acylase	n.s.	n.s.		
		Thermolysin	<0.001 (t = 17.54)	<0.001 (t = 17.68)	<0.001 (t = 17.71)	
		Carboxypeptidase A	<0.001 (t = 408.6)	<0.001 (t = 408.8)	<0.001 (t = 408.8)	<0.001 (t = 391.1)
	Polymyxin B	His$_6$-OPH	n.s.			
		Penicillin acylase	n.s.	n.s.		
		Thermolysin	<0.001 (t = 18.09)	<0.001 (t = 18.33)	<0.001 (t = 18.33)	
		Carboxypeptidase A	<0.001 (t = 84.61)	<0.001 (t = 84.85)	<0.001 (t = 84.86)	<0.001 (t = 66.52)
	Indolicidin	His$_6$-OPH	<0.001 (t = 193.5)			
	Temporin A	His$_6$-OPH	<0.001 (t = 73.75)			
Pseudomonas sp.	Polymyxin E	His$_6$-OPH	<0.001 (t = 21.51)			
		Penicillin acylase	<0.001 (t = 20.36)	n.s		
		Thermolysin	<0.001 (t = 60.83)	<0.001 (t = 82.34)	<0.001 (t = 81.18)	
		Carboxypeptidase A	<0.001 (t = 11.71)	<0.001 (t = 33.22)	<0.001 (t = 32.07)	<0.001 (t = 49.11)
	Polymyxin B	His$_6$-OPH	<0.001 (t = 6.068)			
		Penicillin acylase	<0.001 (t = 6.430)	n.s		
		Thermolysin	n.s	0.011 (t = 3.767)	0.008 (t = 4.128)	
		Carboxypeptidase A	<0.001 (t = 55.03)	<0.001 (t = 61.10)	<0.001 (t = 61.46)	<0.001 (t = 57.34)
	Indolicidin	His$_6$-OPH	<0.001 (t = 24.94)			
	Temporin A	His$_6$-OPH	<0.001 (t = 50.92)			

Table S3. p-values of pairwise multiple comparisons (Holm-Sidak method) after one-way ANOVA of the binding energies (N = 6) of AMPs with different peptidases (Table 4). n.s. – statistically not significant (i.e. p>0.05).

Antimicrobial agent	Enzyme	*vs.* Coccolysin	*vs.* Griselysin	*vs.* Stearolysin	*vs.* Mycolysin	*vs.* Microcystinase
Dermicidin	Griselysin	0.025 (t = 3.030)				
	Stearolysin	<0.001 (t = 4.465)	<0.001 (t = 7.494)			
	Mycolysin	<0.001 (t = 4.943)	n.s.	<0.001 (t = 9.407)		
	Microcystinase	n.s.	n.s.	<0.001 (t = 6.856)	n.s.	
	Carboxypeptidase A	<0.001 (t = 9.726)	<0.001 (t = 12.756)	<0.001 (t = 5.262)	<0.001 (t = 14.669)	<0.001 (t = 12.118)
Polymyxin B	Griselysin	n.s.				
	Stearolysin	<0.001 (t = 7.597)	<0.001 (t = 5.878)			
	Mycolysin	0.002 (t = 4.160)	n.s.	0.009 (t = 3.437)		
	Microcystinase	0.022 (t = 2.984)	<0.001 (t = 4.703)	<0.001 (t = 10.581)	<0.001 (t = 7.145)	
	Carboxypeptidase A	0.006 (t = 3.617)	<0.001 (t = 5.336)	<0.001 (t = 11.214)	<0.001 (t = 7.778)	n.s.
Polymyxin E	Griselysin	n.s.				
	Stearolysin	<0.001 (t = 4.995)	0.001 (t = 4.246)			
	Mycolysin	n.s.	n.s.	<0.001 (t = 6.494)		
	Microcystinase	n.s.	n.s.	<0.001 (t = 4.912)	n.s.	
	Carboxypeptidase A	<0.001 (t = 6.994)	<0.001 (t = 7.743)	<0.001 (t = 11.989)	<0.001 (t = 5.495)	<0.001 (t = 7.077)
Oritavancin	Griselysin	n.s.				
	Stearolysin	<0.001 (t = 8.704)	<0.001 (t = 8.777)			
	Mycolysin	<0.001 (t = 4.497)	0.001 (t = 4.425)	<0.001 (t = 13.201)		
	Microcystinase	n.s.	n.s.	<0.001 (t = 9.792)	0.015 (t = 3.409)	
	Carboxypeptidase A	n.s.	n.s.	<0.001 (t = 11.098)	n.s.	n.s.
Indolicidin	Griselysin	<0.001 (t = 12.372)				
	Stearolysin	<0.001 (t = 13.362)	<0.001 (t = 25.733)			
	Mycolysin	<0.001 (t = 5.196)	<0.001 (t = 7.176)	<0.001 (t = 18.558)		
	Microcystinase	n.s.	<0.001 (t = 13.238)	<0.001 (t = 12.496)	<0.001 (t = 6.062)	
	Carboxypeptidase A	<0.001 (t = 10.269)	n.s.	<0.001 (t = 23.630)	<0.001 (t = 5.072)	<0.001 (t = 11.135)
Temporin A	Griselysin	n.s.				
	Stearolysin	<0.001 (t = 5.565)	<0.001 (t = 5.008)			
	Mycolysin	<0.001 (t = 5.120)	<0.001 (t = 4.563)	n.s.		
	Microcystinase	<0.001 (t = 8.347)	<0.001 (t = 7.791)	0.045 (t = 2.782)	0.021 (t = 3.228)	

	Carboxypeptidase A	n.s.	n.s.	0.013 (t = 3.450)	0.032 (t = 3.005)	<0.001 (t = 6.232)
